# Factor XII Deficiency Mimicking Bleeding Diathesis: A Unique Presentation and Diagnostic Pitfall

**DOI:** 10.7759/cureus.2817

**Published:** 2018-06-15

**Authors:** Hermina D Fernandes, Shauna Newton, Jonathan M Rodrigues

**Affiliations:** 1 Internal Medicine, University of North Dakota School of Medicine and Health Sciences, Bismarck, USA

**Keywords:** factor xii, factor xii deficiency, factor xii deficiency, activated partial thromboplastin time, coagulation, blood coagulation, coagulation factors, hageman factor

## Abstract

Factor XII (FXII), also known as Hageman factor, is a coagulation protein that is necessary for the functioning of the intrinsic coagulation cascade and fibrin formation. When deficient, it results in a significant prolongation of activated partial thromboplastin time (aPTT), mimicking a bleeding disorder. However, it does not result in clinical bleeding tendency. We report a case of an elderly male who was found to have prolonged aPTT, discovered during preoperative evaluation for operative repair of hip fracture. Although laboratory investigation was suggestive of bleeding tendency, he was diagnosed with factor XII deficiency and had no bleeding complications intra-operatively or in the post-operative period.

## Introduction

Coagulation factor XII (FXII) is a single chain glycoprotein, present in plasma as the zymogen of serine protease factor XIIa (FXIIa). It plays a primary role in the initiation of the intrinsic coagulation cascade and fibrin formation [1-2]. FXII deficiency is a rare, autosomal recessive disorder, with an incidence of approximately 1/1,000,000 individuals [3]. Although it results in a marked prolongation of activated partial thromboplastin time (aPTT), FXII deficiency does not result in bleeding disorders [2]. This is distinct from deficiencies of other coagulation factors of the coagulation cascade that do result in bleeding disorders, such as factor VIII or factor IX (that cause Hemophilia A and B, respectively), as well as deficiencies of FVII and, tissue factor (TF) [1].

FXII deficiency is most often incidentally discovered [2]. It causes a severely prolonged aPTT, which is an in-vitro coagulation test that suggests bleeding tendency. However, in contrast, it is associated with preserved hemostasis in-vivo. FXIIa plays a role in the direct activation of plasmin into plasminogen [1,3]. Today, congenital FXII deficiency is suggested by the presence of an isolated elevation in aPTT after other causes have been ruled out [3].

## Case presentation

A 75-year-old Caucasian male presented to the hospital after a fall and was diagnosed with a left hip intertrochanteric femur fracture secondary to trauma. He was not on any prescription medication including oral anticoagulants. His prior surgical history included an uncomplicated tonsillectomy in childhood. He did not endorse a past history of prolonged bleeding or thrombotic episodes. He had no documented history of liver disease, significant alcohol use, dyspnea, or painful swollen extremities. On exam, he was breathing comfortably at rest, had a normal heart rate, and had no peripheral extremity edema. The left hip was externally rotated, sensation was intact in the extremity, and the foot was well perfused. Plain radiographs of the left hip revealed an intertrochanteric fracture of the left proximal femur. Operative repair of hip fracture was planned.

Routine preoperative laboratory testing revealed a hemoglobin of 9.7g/dL (normal range 14.0 - 18.0 g/dL) and hematocrit of 27.9% (normal range 37.0% - 52.0%), prolonged aPTT at 61 seconds (normal range 22-29 seconds). Prothrombin time (PT) was normal at 10.8 seconds (normal range 9.7-11.9 seconds). Mild transaminitis was noted after fall with alanine aminotransferase (ALT) 62 U/L (normal range <40 U/L) and aspartate aminotransferase (AST) 121 U/L (normal <35 U/L). Gamma-glutamyl transpeptidase (GGTP) was within normal limits at 10 U/L (normal range <60 U/L), ruling out acute ethanol consumption. Total bilirubin was normal at 1.1 mg/dL (normal range <1.2 mg/dL), alkaline phosphatase was normal at 35 U/L (normal range 35-130 U/L). Ethanol and acetaminophen blood levels were undetectable and an ultrasound examination of the liver did not demonstrate any liver disease. Serology for viral hepatitis A, B, and C was normal. Transaminitis improved and was subsequently normal. However, aPTT was persistently elevated on multiple measurements. Disseminated intravascular coagulation was ruled out with negative testing for fibrin monomer, normal PT and no evidence of low fibrinogen level. Fibrinogen at 568 mg/dL (normal range 185-408 mg/dL) and mildly elevated D dimer at 6.92 mg/L FEU (normal range <0.53 mg/L FEU) reflected their nature as acute phase reactants. Reptilase time was normal at 17 seconds (normal range 14-23 seconds), ruling out spurious heparin contamination in the intravenous line. Thrombin time was 14 seconds and on the lower end of normal reference range of 15-23 seconds. This was of no known hemostatic consequence. Normal dilute Russell viper venom time (dRWT) with ratio 1.1 (normal reference range 0.0-1.1) suggested no evidence of lupus anticoagulant. Mixing study of prolonged aPTT demonstrated no inhibition and, therefore, no evidence of lupus anticoagulant. Antiphospholipid antibody immunoglobulin G (IgG) and immunoglobulin M (IgM) as well as beta-2 glycoprotein antibody IgG and IgM were normal (<9.4 MPL with normal reference range <15MPL, <9.4MPL with reference range <15MPL, <6.4CU with normal reference range <20CU, <1.1 CU with normal reference range <20CU, respectively).

The lack of painful or swollen extremities to suggest a venous or arterial thromboembolic event along with his laboratory results made a diagnosis of antiphospholipid syndrome unlikely. The data from mixing study reflected no inhibition and suggested a coagulation factor deficiency, as the patient had correction immediately and after incubation at 37 ^o^C. Assays of factors II, V, VIII, IX, X, XI, and XII along with von Willebrand factor assay were performed. Factor VIII activity assay was elevated at 205%, along with elevated von Willebrand factor antigen (vWFAg) at 322% and elevated von Willebrand factor activity (vWFActivity) at 358% (normal reference range 55%-200%). Levels of factor VIII, vWFAg and vWF activity were concordant, reflecting their activity as acute phase reactants.

Factor II assay was normal at 86% (normal reference range 75%-145%). Factor V assay was normal at 94% (normal reference range 70%-165%). Factor X assay was normal at 104% (normal reference range 70%-150%). Factor XI assay was normal at 102% (normal reference range 55%-120%). Factor IX assay was 159.9% (normal reference range 65%-140%) and likely represented acute phase reactant. Since the patient’s plasma had normal or elevated coagulation factor activity, no inhibitor screen was performed for specific inhibitors to factor II, V, VIII, IX, X, XI. Factor XII assay was 2% and confirmed a coagulation factor deficiency with a marked reduction in factor XII activity, well below the reference range of 55%-180%. Therefore, the patient was diagnosed with factor XII or Hageman factor deficiency.

Since this deficiency is of no known hemostatic consequence, the patient proceeded with surgery. A left hip trochanteric fixation was performed without complications. At the time of his hospitalization and treatment, the literature regarding the increased risk of venous thromboembolism in this condition remained uncertain. Therefore, he received extended duration postoperative prophylaxis with 40 mg of subcutaneous enoxaparin daily for three weeks after surgery [4]. Low dose aspirin 81 mg oral daily was prescribed after discontinuation of enoxaparin for prevention of venous thromboembolism [5]. At his four-month post-surgical clinic follow up, the patient reported no bleeding or thromboembolic events.

## Discussion

The aPTT represents a laboratory assessment of the status of the intrinsic and final coagulation pathway. It measures the time for human plasma to clot, in seconds. Prolongation of aPTT occurs with deficiencies or inhibitors of the intrinsic and final coagulation pathway [6].

A prolonged aPTT should prompt further investigation especially in the setting of no anticoagulant use and no prior liver disease. Other etiologies that prolong aPTT such as elevated hematocrit, incomplete collection tube filling, line contamination by anticoagulants, prolonged time interval between blood sample collection, and assay performance should be ruled out. Elevated hematocrit >55% may result in less plasma (which contains coagulation factors) and leads to prolonged aPTT. Inadequately filled citrate tubes have less coagulation factors relative to citrate, leading to falsely prolonged aPTT. Factor VIII of the intrinsic pathway is labile and so assays run greater than four hours after specimen collection may lead to falsely prolonged aPTT [6]. After these etiologies are ruled out, a mixing study is performed. Here, a patient’s plasma is mixed with normal plasma at a 1:1 ratio. Subsequently, PT and aPTT are performed immediately and after incubation at 37^o^C. Impaired correction at either assessment indicates the presence of an inhibitor. Correction at both assessments indicates coagulation factor deficiency of one or more coagulation factors. Coagulation factor assays for factors VIII, IX, XI and XII are then performed. Deficiencies of factors VIII, IX, and XI result in the bleeding disorders hemophilia A, hemophilia B, and hemophilia C, respectively. On the other hand, deficiency of FXII does not cause bleeding disorder [6].

Dr. Ratnoff OD and colleagues initially described FXII deficiency in 1955 in a report of three patients presenting with prolonged clotting time of venous blood without hemorrhagic symptoms [7]. None had a history of bleeding or thrombotic episodes despite histories of prior surgeries, menses, or childbirth. This group reached a conclusion that this was due to deficiency of a substance found in normal plasma responsible for normal coagulation.

Fifteen years later, one of the initial three patients, Mr. John Hageman, presented to the hospital after falling from a ladder of a boxcar [8]. He had fractured his left hemipelvis. He was previously noted to have a prolonged clotting time at the age of 37 [8-9]. This abnormality was detected through routine lab tests in preparation for a partial gastrectomy and gastrojejunostomy, due to persistent peptic ulcer disease [8]. His past surgical history included a tonsillectomy and dental extraction, neither of which resulted in excessive bleeding [9]. His surgery was performed without complications. Treatment at the time included bedrest for one week and physiotherapy, which allowed Mr. Hageman to walk with crutches. On the twelfth day of hospitalization, Mr. Hageman died from a large saddle pulmonary embolus [8]. At the time, FXII deficiency was thought to confer elevated thrombotic risk. However, in this instance, the association is debatable, since it is now known that this patient had risk factors for pulmonary embolism independent of FXII deficiency, including a hip fracture and post-surgical immobilization. Factor XII was subsequently named Hageman factor.

However, several studies from 1983-1993 have suggested that FXII deficiency may increase the risk of thrombosis. A report by Goodnough et al. [10] in 1983 identified 121 patients with Hageman trait (less than 1% FXII activity). Ten (8%) had reported a history of thrombosis, five had a history of myocardial infarction, and one had a history of Moyamoya disease. None of these patients had a history of bleeding tendency. Another case report in 1984 [11] described the occurrence of myocardial infarction in a 40-year old man with severe FXII deficiency (activity <1%) without cardiac risk factors. This patient’s blood pressure was 140/90, and his glucose and lipid profile tests were all within normal range. In 1991, a study by Lämmle et al. of 74 subjects from Swiss families with FXII deficiency suggested that homozygous FXII deficiency is associated with an increased risk for venous thrombosis, but partial FXII deficiency is not [2]. In this study, two out of 18 subjects (11%) with homozygous FXII deficiency had deep vein thrombosis at an age of less than 40 years, while only one among the 45 subjects (2%) with heterozygous FXII deficiency had a history of venous thrombosis. The data from this study also suggests the absence of a bleeding diathesis in FXII deficiency.

More recently, animal model research studies have sought to refine our knowledge of this clinical entity. Research on murine knockout models for FXII has demonstrated normal hemostasis in the presence of defective thrombus formation, with protection from cerebral ischemia and pulmonary embolism [1]. FXII deficient humans also do not demonstrate a bleeding tendency in the presence of a prolonged aPTT [1].

FXII plays a vital role in the contact activation pathway where it activates factor XI to factor XIa and prekallikrein to kallikrein [12]. It also activates plasminogen to plasmin in the fibrinolytic pathway. The resultant cascade leads to the development of bradykinin-mediated angioedema. C1 inhibitor is a major inhibitor of contact system proteases, including factor XIIa, and inhibits the fibrinolytic protease plasmin. C1-esterase inhibitor deficiency leads to a disproportionate activation of the FXII-driven contact system cascade and the development of edema in hereditary angioedema (HAE) type I and II patients. A third type of familial angioedema exists in which patients have normal antigenic and functional C1 inhibitor levels. These patients are classified as hereditary angioedema (HAE) with normal C1-inhibitor levels, formerly known as HAE type III. Some, but not all, of these patients have a gain-of-function mutation in FXII [12-13].

FXII has also been demonstrated to be activated by poly P, an inorganic phosphate polymer substance that exists on human platelet dense granules. Studies also show that poly P is linked to bacterial growth and survival. This suggests a role for platelet poly P-mediated FXII activation among the defense mechanisms in infection as well as inflammation and thrombosis [9].

## Conclusions

FXII deficiency is a rare cause of elevated aPTT. Despite a prolongation of coagulation time, this deficiency does not result in bleeding diathesis. While research to date has shown that FXII deficiency does not result in bleeding tendency, its role as a thrombotic risk factor is still debatable. Due to its infrequent occurrence, no large-scale studies have been performed. However, because of its role in thrombosis without an effect on hemostasis, researchers have proposed that antibodies targeting FXII may be of antithrombotic benefit without affecting hemostasis.
